# Data related to influence of process parameters on the microstructure, structural and mechanical properties of additive manufactured titanium alloy composites

**DOI:** 10.1016/j.dib.2022.108181

**Published:** 2022-04-18

**Authors:** Yonas Tibebu Mekonnen, Endelkachew Addis Mekonen, O. Fatoba

**Affiliations:** Department of Mechanical Engineering, Debre Tabor University, Debre Tabor, Ethiopia

**Keywords:** Ti6Al4V, Mechanical properties, Quaternary coatings, DLMD, Microstructure, Process parameters

## Abstract

Instead of tampering with the whole microstructure of titanium alloy base structure, direct laser metal deposition (DLMD) method can be used to target and solve a particular problem. This will result in extended application of titanium alloy. The enhancement of titanium alloy service life span can be done by fabricating composite coatings on titanium alloy. Additive manufacturing technique has been used over the years to repair and extend components’ life span. Experimental procedure was done at the National Laser Center, CSIR, South Africa with Ytterbium Laser System inbuilt with 3000 W for the quaternary coatings. Materials characterization was done according to the standard procedure. The stable beta phase in the copper reinforcement is very strong and this phase is propagated during the direct laser metal deposition process. The initiation and propagation of phases of beta-titanium structures (β-Ti) was due to the reaction in the molten pool of reinforcement copper and titanium alloy base. Also, aluminium-copper structures formed and brought up dendritic grain propagation as a result of feed rate of the reinforcement power, laser power and scanning velocity increase. The significant role played by the aluminium reinforcement in the lattice structures led to the titanium-aluminides (TiAl) propagation. The visibility of the dendritic phases as seen in the microstructures came about as aluminium reinforcement reacts with copper in the matrix as the molten pool solidifies. The properties were enhanced as a result of optimized process parameters.

## Specification Table

**Table:** 

Subject area	Metals and Alloy, Thermal Engineering and Additive Manufacturing
Specific subject area	Influence of Optimized Process Parameters on the Properties of Additive Manufactured Titanium Alloy Composites
Type of data	Figures, Tables, Graphs and Images
Description of data collection	Ti-Al-Si-Cu as follows: Ti-Al-11Si-5Cu (900 W, 1.0 m/min), Ti-Al-11Si-5Cu (1.2 m/min, 900 W), Ti-Al-9Si-3Cu (1.0 m/min, 1000 W), Ti-Al-9Si-3Cu (1.2 m/min, 1000 W), Ti-Al-7Si-1Cu (1.0 m/min, 1000 W), Ti-Al-7Si-1Cu (1.2 m/min, 1000 W), Ti-Al-12Si-2Cu (1.0 m/min, 900 W), Ti-Al-12Si-2Cu (1.2 m/min, 900 W), Ti-Al-13Si-6Cu (1.0 m/min, 900 W), Ti-Al-13Si-6Cu (1.2 m/min, 900 W), Ti-Al-7Si-4Cu (1.0 m/min, 1000 W), Ti-Al-7Si-4Cu (1.2 m/min, 1000 W) were used for the reinforcements for the quaternary composite coatings. Oxidation is not needed during the experimental procedure, so the gas (Argon) shield rate eliminates it. Argon was chosen because is cheaper than other gases. During the direct laser metal deposition (DLMD) procedure, over lapping of tracks were done at 70%. The base material distance to the tip of the nozzle was maintained at 2 mm. The procedure was performed in South Africa at National Laser Center (NLC, CSIR. 3000 W Ytterbium Laser System (YLS) was used throughout the experiment for ternary composite coatings. Though minutes pores were observed, but the optimized parameters proved microstructures homogenous of the samples. With different paper sizes, grinding and polishing were done on the coated samples. All the samples showed mirror like surfaces after polishing with diamond suspension and were rinsed in distilled water and dried in air. Characterization of all the samples considered for both the surface and cross-sectional views of the microstructures. All the microstructural images of the samples were done via Olympus Microscope (BX51M) and TESCAN-X-MAX scanning electron microscopy. The hardness results were taken from Vickers Microhardness Tester. The parameters for the experimental procedure were optimized via design of experiment. The following parameters were optimized: powder feed rate (2.5 g/min), gas shield rate (2.5 L/min), velocity of scanning (1.0 −1.2 m/min), and laser power (900–1000 W). Oxidation is not needed during the experimental procedure, so the gas (Argon) shield rate eliminates it. Argon was chosen because is cheaper than other gases. During the direct laser metal deposition (DLMD) procedure, over lapping of tracks were done at 70%. The base material distance to the tip of the nozzle was maintained at 2 mm. The procedure was performed in South Africa at National Laser Center (NLC, CSIR. 3000 W Ytterbium Laser System (YLS) was used throughout the experiment for the quaternary composite coatings
Data Format	Raw, analyzed
Data source location	Department of Mechanical Engineering, Debre Tabor University, Debre Tabor, Ethiopia.
Data Availability	Data are available within this article.

## Value of the Data

• Compared to conventional techniques, this data has shown that using direct metal laser deposition (DLMD) additive manufacturing technique can enhance the surface integrity of Titanium alloy (Ti6Al4V) with extended application in the aerospace industry. The manufacturing of Novel composite coatings is possible as indicated in this research. This novel composite coatings data is viable for applications in aerospace industry because of good metallurgical properties, enhanced mechanical and structural performance.
• Aluminium-copper structures formed and brought up dendritic grain propagation as a result of feed rate of the reinforcement power, laser power and scanning velocity increase. Copper as a reinforcement had been reported to have strong stabilized beta (β) particles. Likewise, beta-titanium phase (β-Ti) propagated as a result of the fusion of titanium alloy and copper reinforcement. Moreover, intermetallic inter-dendritic eutectic phases network was formed by the combined beta and alpha phase of copper and aluminium reinforcements. These results would help materials engineer, Thermal engineers, and metallurgical engineers to understand the metallurgical evolutions of this quaternary composites.
• When the process parameters are optimized, the quaternary composite coatings can be used by direct laser metal deposition technique to enhance the microstructures, mechanical properties and surface integrity of Titanium alloy for aerospace application. These data would help engineers in the aerospace industry to understand the influence of optimized process parameters on the metallurgical evolution.
• One of the visible phases in the microstructure is Titanium-aluminide compounds which is responsible for the enhancement in hardness property. The composition of the novel composite coating also attributes to the enhancement of the surface integrity. Increase in Hardness property (528 HV) by 32.2% and 32.13% increase in the ultimate tensile strength (5179 MPa) came about as a result of the optimized process parameters at scan velocity of 1.2 m/min and laser power of 900 W. Silicon was at 13 wt% and Copper at 6 wt% (Ti-Al-Si-Cu) to achieve this enhancement. These data help metallurgical engineer to understand quaternary Ti-Al-Si-Cu composites for various application in aerospace industry.

## 1 Data Description

**Fig. 1:**
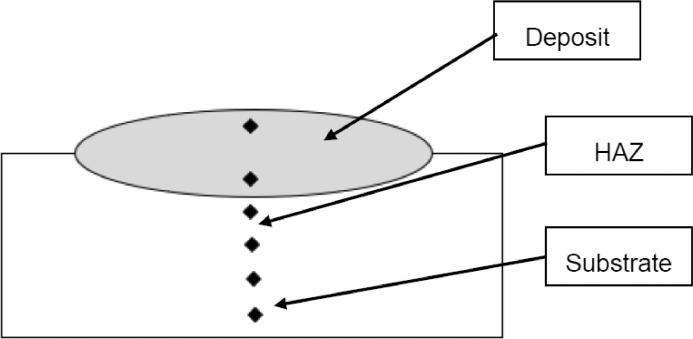
Cross section indentation.

**Fig. 2:**
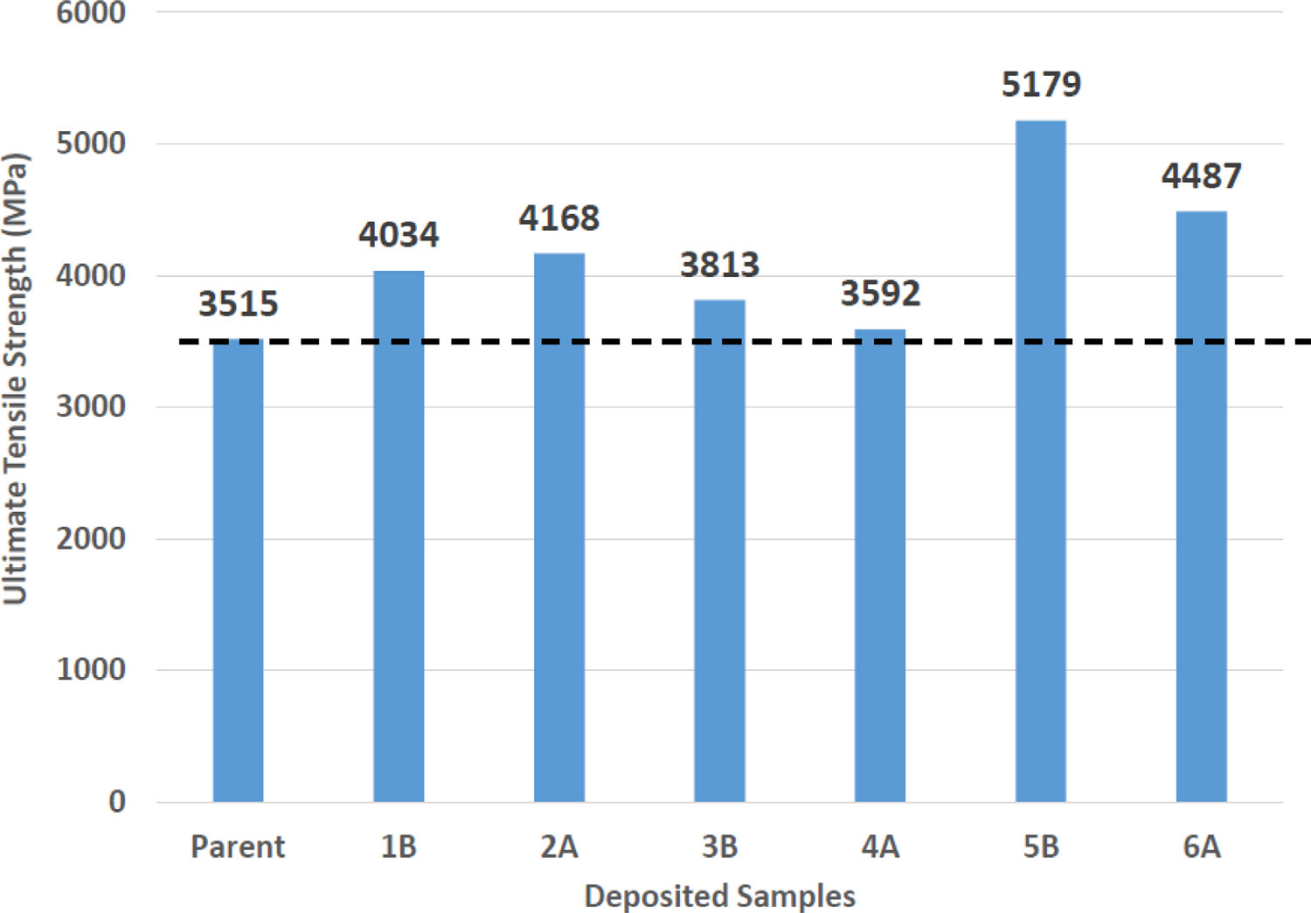
Comparison of quaternary composite coatings UTS and the base metal.

**Fig. 3:**
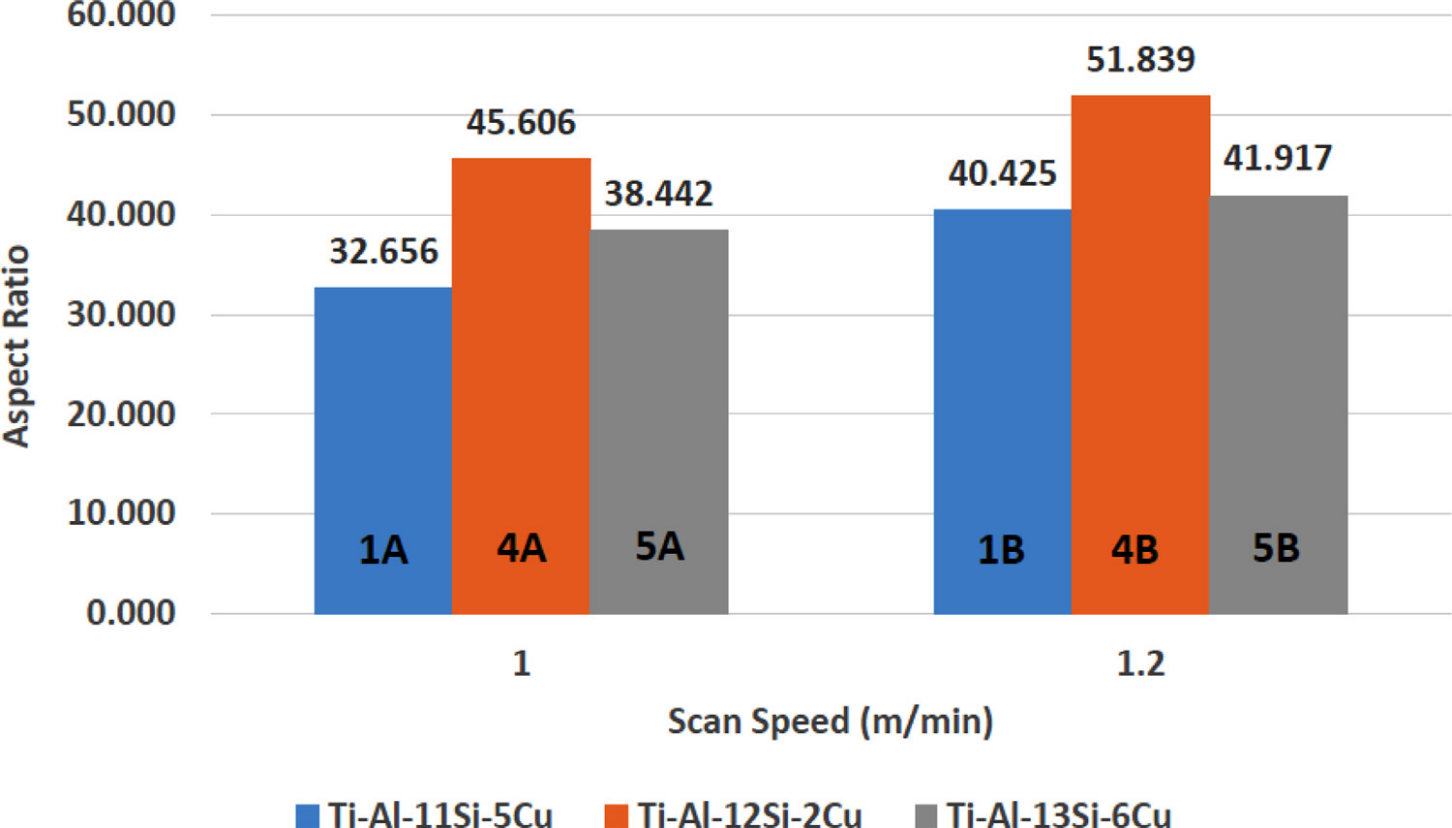
Quaternary composite coatings aspect ratio at 900 W.

**Fig. 4:**
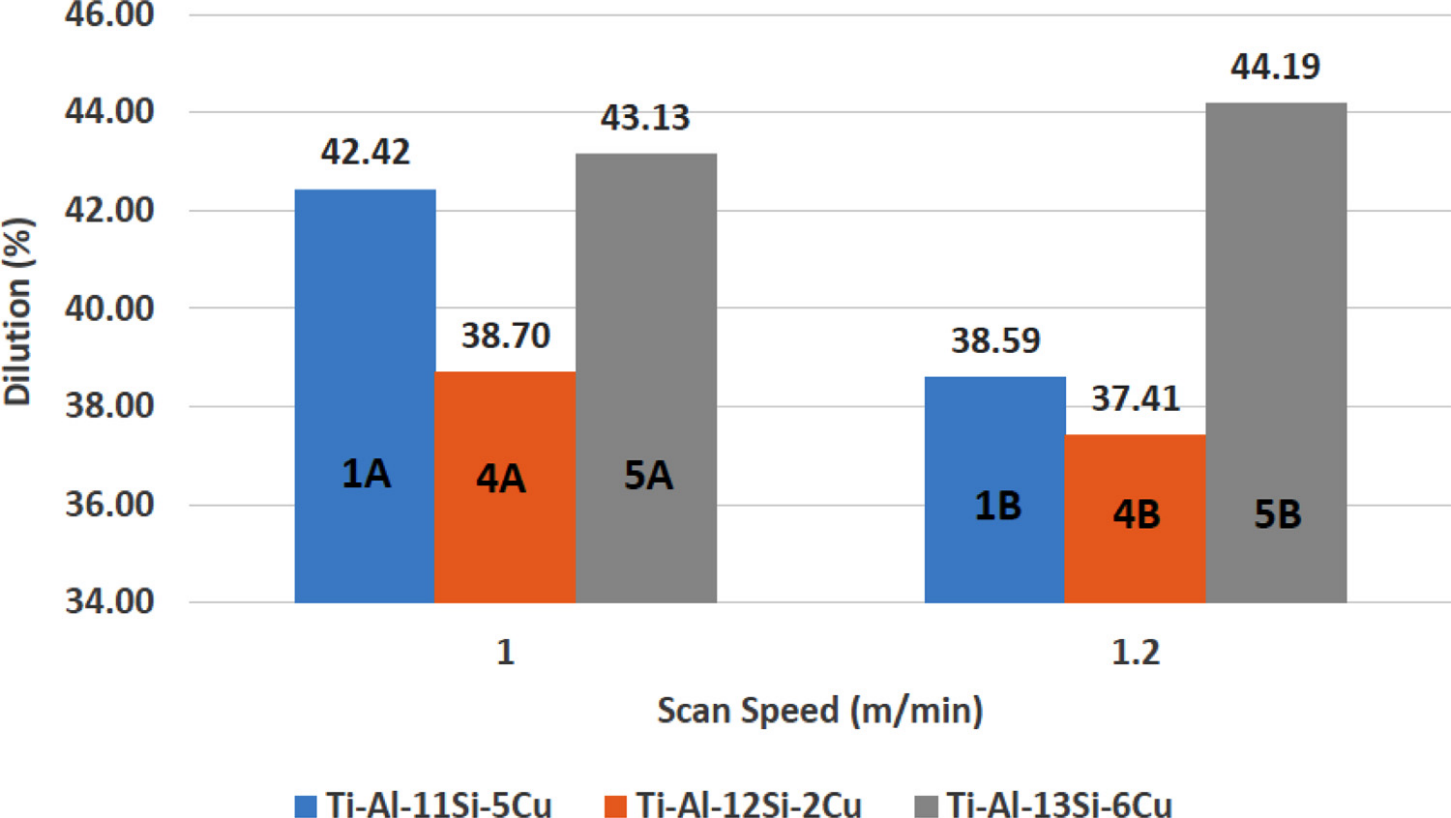
Quaternary composite coatings dilution at 900 W.

**Fig. 5:**
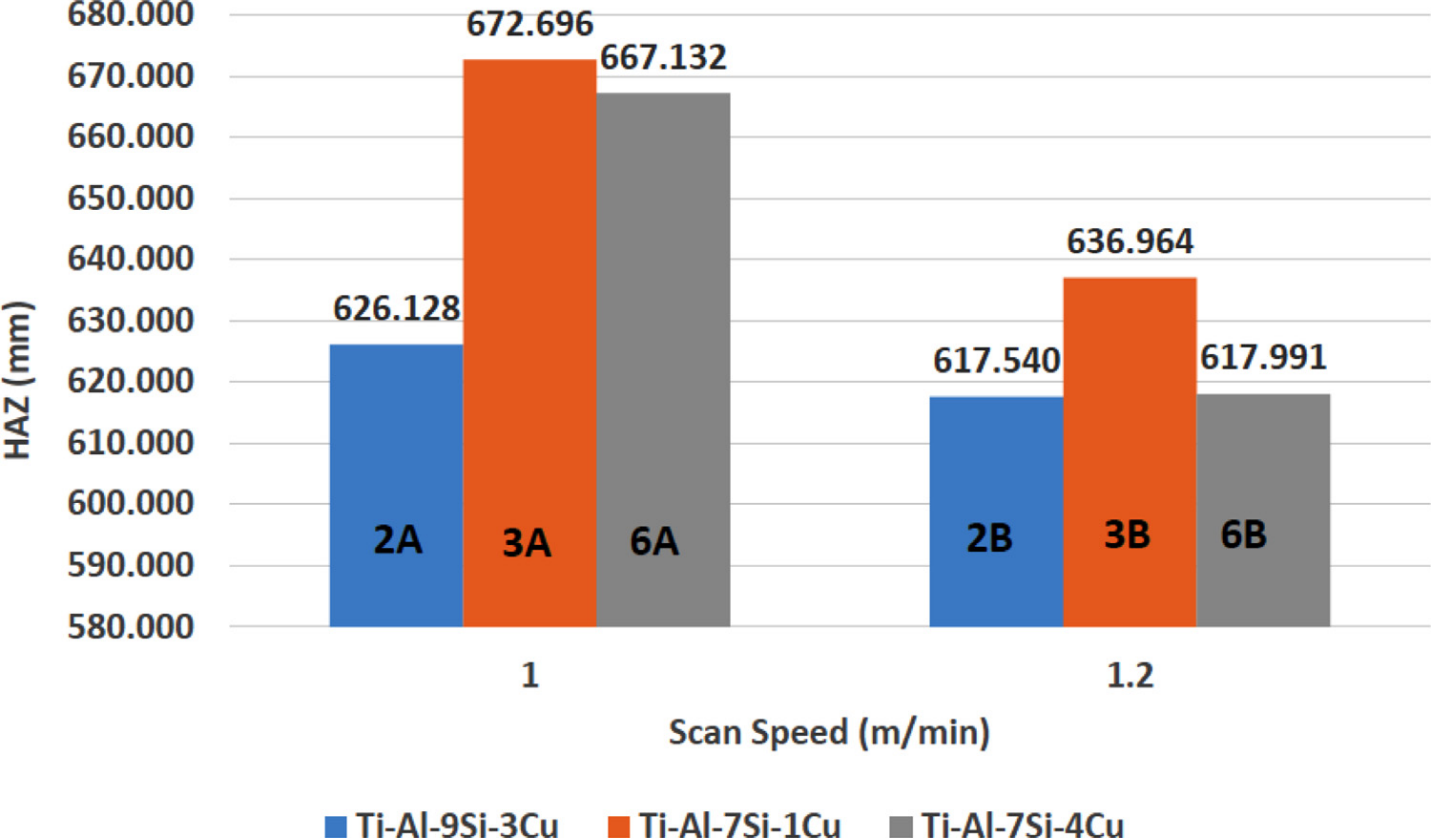
Heat affected zone of quaternary composite coatings at 900 W.

**Fig. 6:**
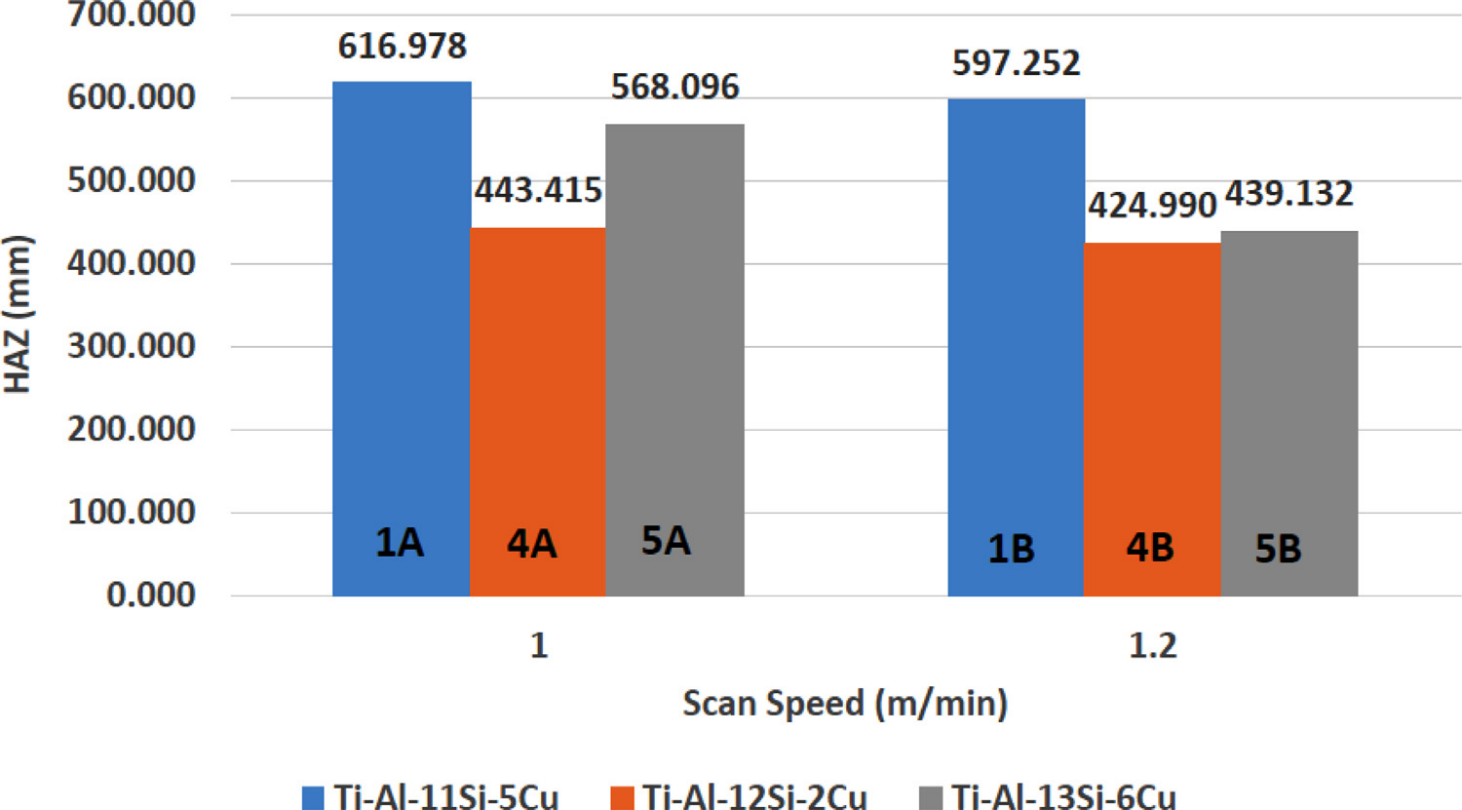
Heat affected zone of quaternary composite coatings at 1000 W.

**Fig. 7:**
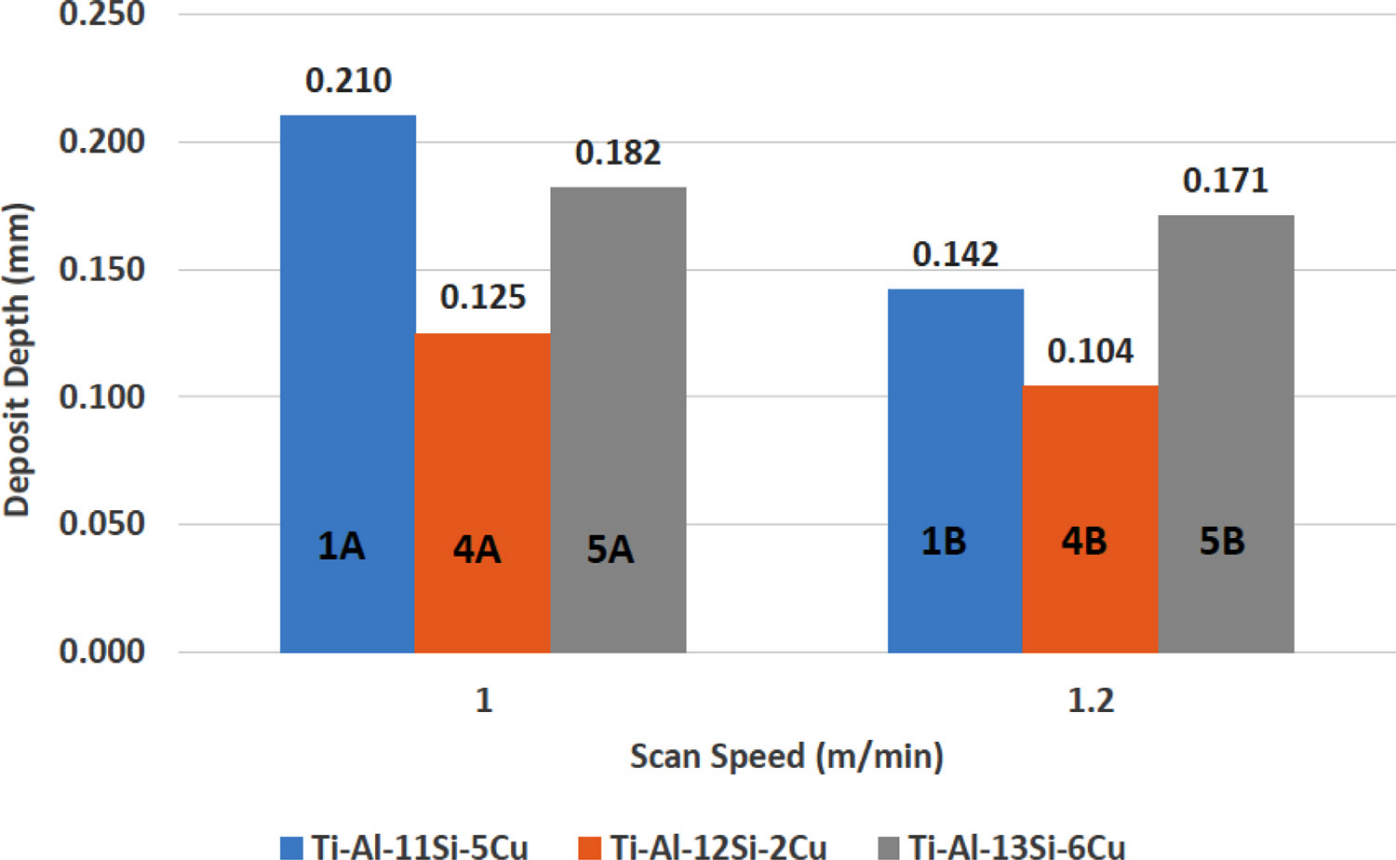
Deposit depth of quaternary composite coatings at 900 W.

**Fig. 8:**
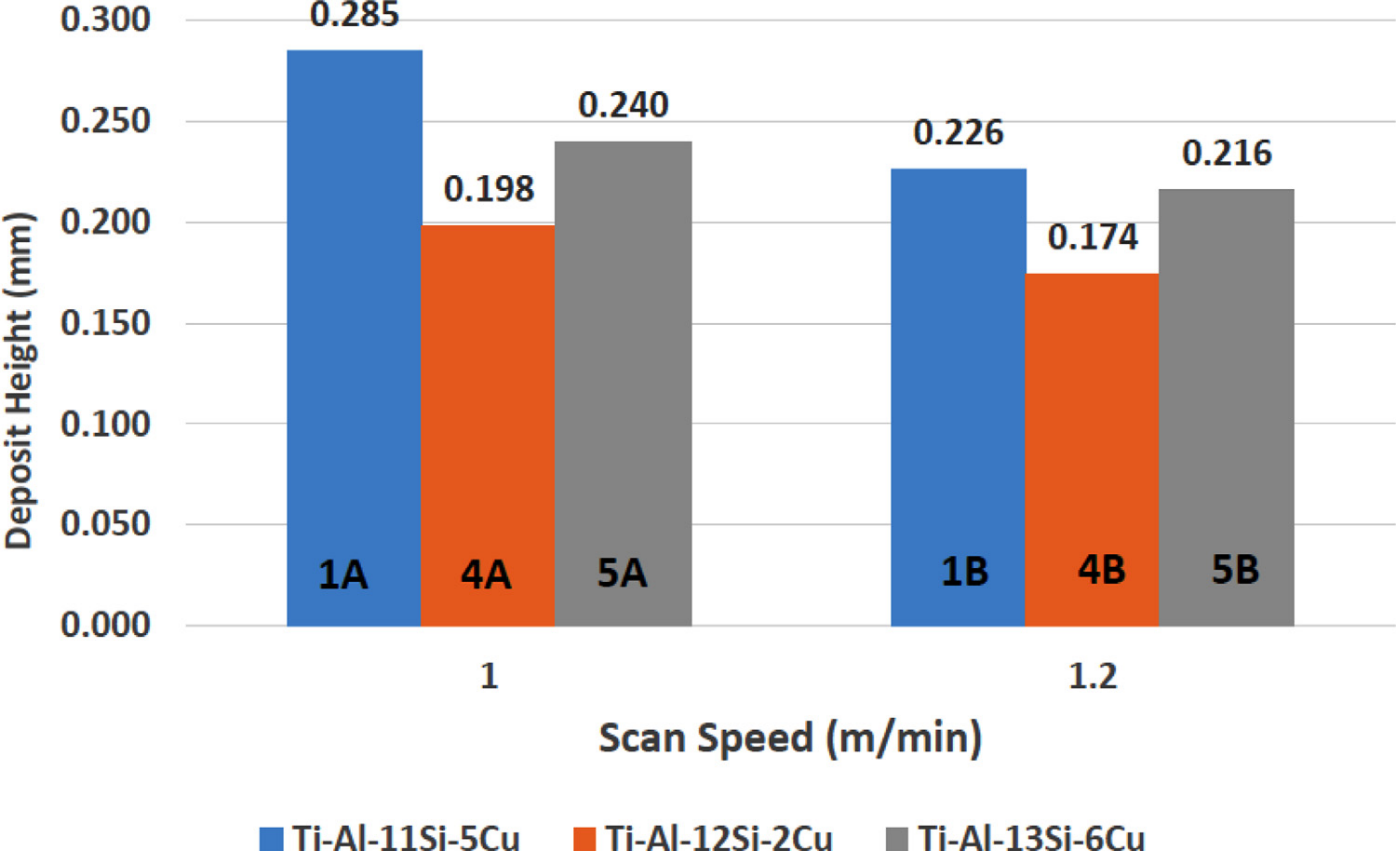
Deposit height of quaternary composite coatings at 1000 W.

**Fig. 9:**
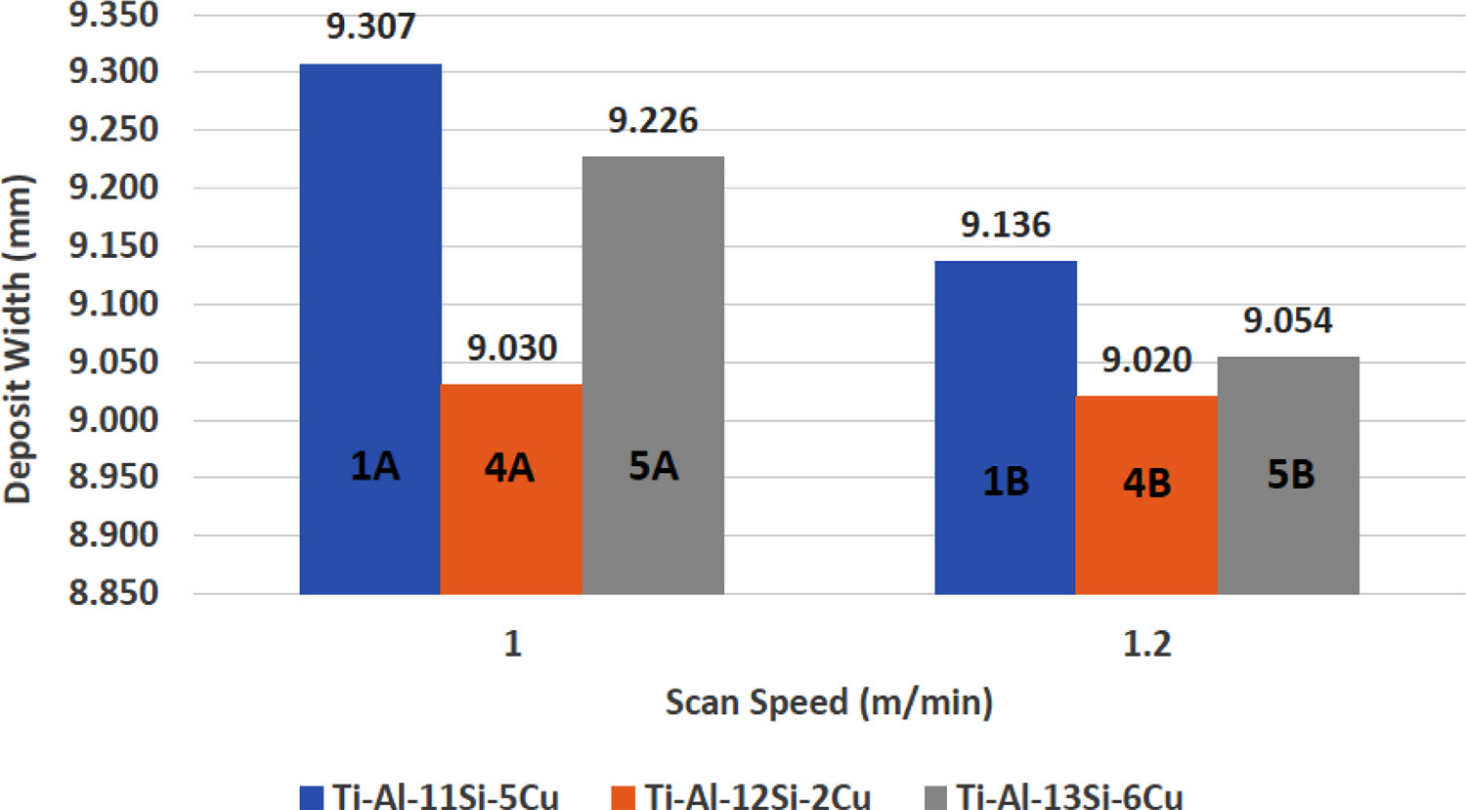
Deposit width of quaternary composite coatings at 1000 W.

**Fig. 10:**
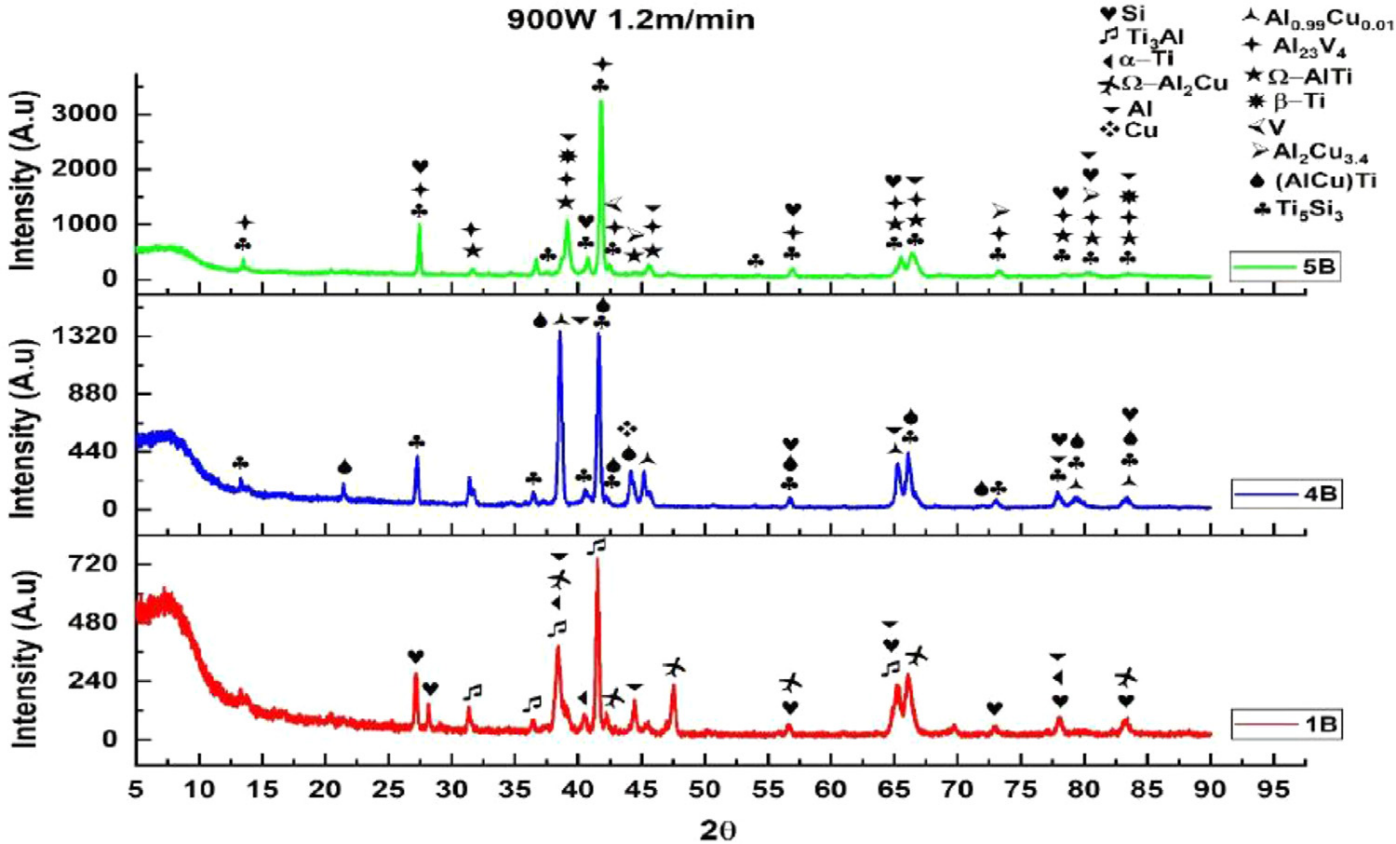
XRD of quaternary composite coatings at 900 W and scan velocity of 1.2 m/min.

**Fig. 11:**
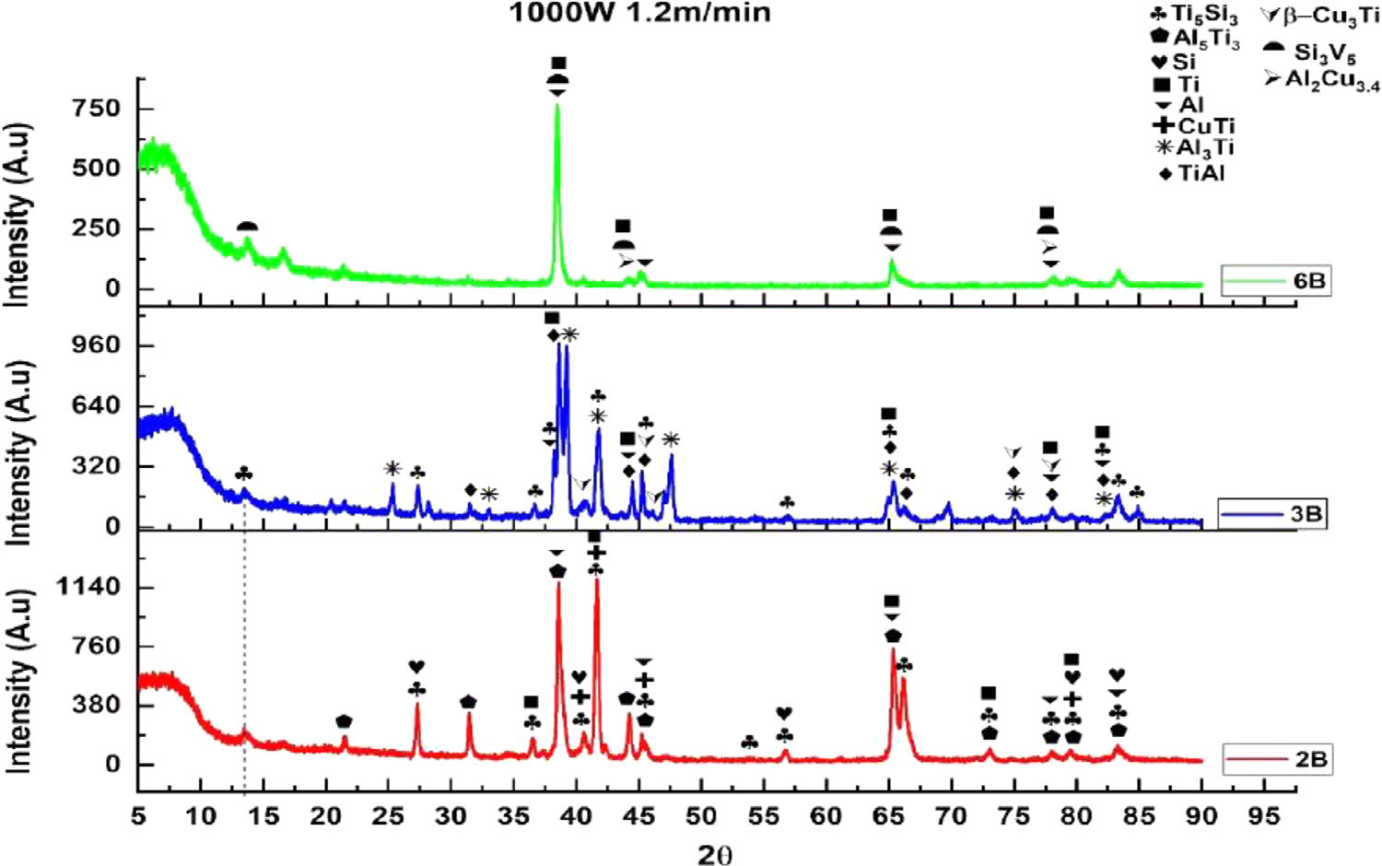
XRD of quaternary composite coatings at 1000 W and scan velocity of 1.2 m/min.

**Fig. 12:**
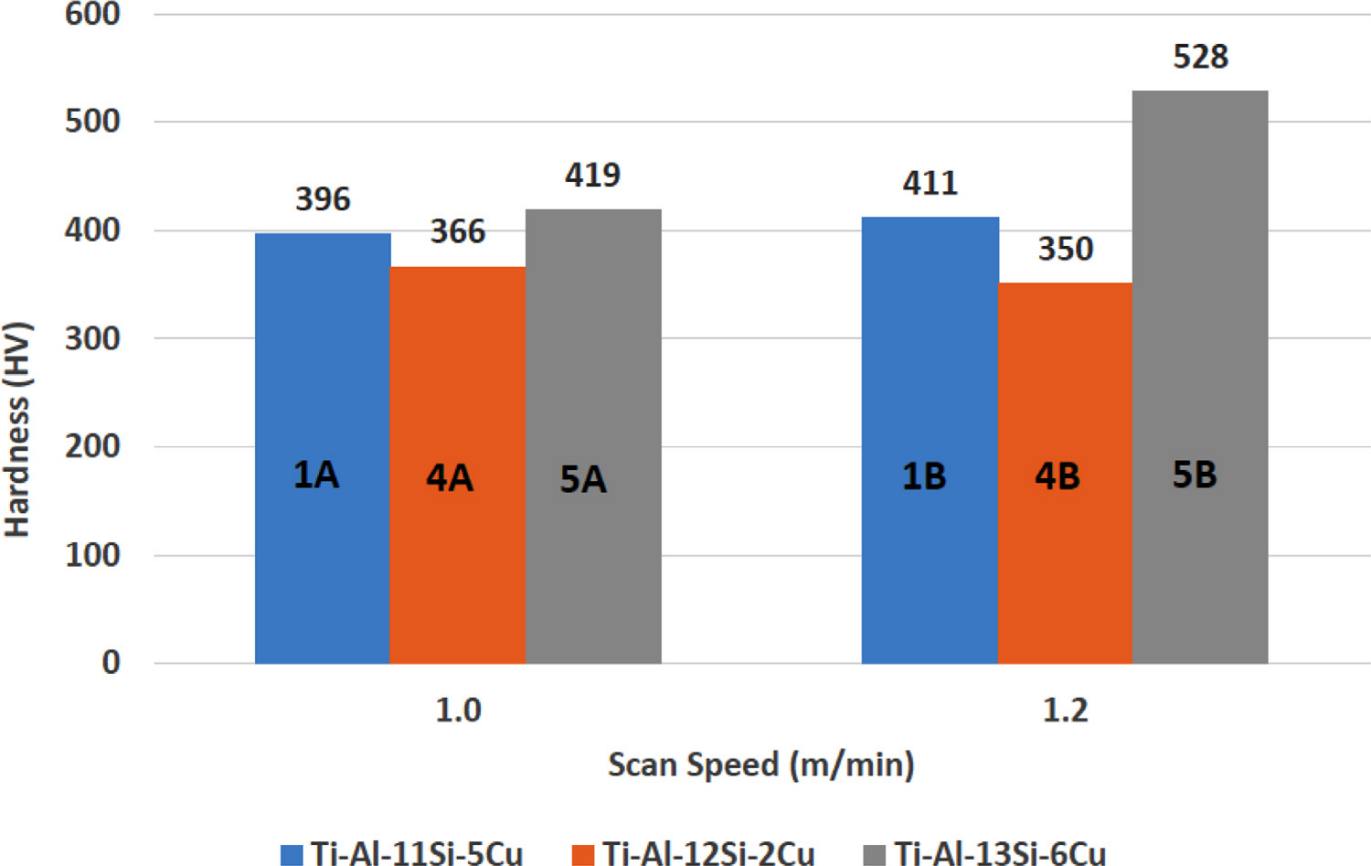
Hardness property of quaternary composite coatings at 900 W.

Fig. 1 shows how the indentation was done during the hardness testing. Each sample were indented 5 times and the average values became the final hardness values. Table 1 shows the tabulation of calculated laser energy density and laser materials interaction time. The quantity of composite coatings that can be done unto the base metal is determined by the processing energy magnitude in J/mm^2^. This is linked to the scan velocity and the laser power. Table 2 gives the quaternary composite structural properties in terms of heat affected zone (HAZ), deposit height, deposit width, aspect ratio, dilution and depth. While Table 3 gives the hardness property of the quaternary composite coatings. Table 4 also gives the ultimate tensile strength (UTS) of the composite coatings. Fig. 2 did comparison between the base metal (Titanium alloy) ultimate tensile strength and the UTS of the quaternary composite coatings. There was increase of 32.13% as compared to the base metal. The aspect showed in Fig. 3 decreased with increase in the weight percent of Silicon in the composite coating at 900 W. Decrease in dilution was noticed in the samples manufactured at 900 W as seen in Fig. 4. There was general decrease in the HAZ for all samples as the scan velocity increases either at 1000 W or 900 W as shown in Figs. 5 and 6. Figs. 7, 8 and 9 show the deposit height, deposit width and deposit depth with increase in scan velocity. The XRD spectra of the coatings re shown in Figs. 10 and 11. All the intermetallic phases and compounds formed during the direct laser metal deposition (DLMD) with the base metal can be seen in these Figures. These phases contribute to the enhancement in the hardness property and microstructures [1], [2], [3], [4], [5]. The hardness property of all the samples is indicated in Fig. 12.

**Table 1:** Tabulation of laser energy density (LED) and laser material interaction time (LMIT).

Powder Composition	Sample ID	Laser Power (W)	Scanning Speed (m/min)	LED (J/mm^2^)	LMIT (s)
Ti-Al-11Si-5Cu	1A	900	1.0	27	3.9
	1B		1.2	22.5	3.25
Ti-Al-9Si-3Cu	2A	1000	1.0	30	3.9
	2B		1.2	25	3.25
Ti-Al-7Si-1Cu	3A	1000	1.0	30	3.9
	3B		1.2	25	3.25
Ti-Al-12Si-2Cu	4A	900	1.0	27	3.9
	4B		1.2	22.5	3.25
Ti-Al-13Si-6Cu	5A	900	1.0	27	3.9
	5B		1.2	22.5	3.25
Ti-Al-7Si-4Cu	6A	1000	1.0	30	3.9
	6B		1.2	25	3.25

**Table 2:** Quaternary composite coatings structural properties.

Powder Composition	Sample ID	Laser Power (W)	Scan Speed (m/min)	Deposit Width (mm)	Deposit Height (mm)	Deposit Depth (mm)	HAZ Height (mm)	Dilution (%)	Aspect Ratio	Area (mm^2^)	Volume (mm^3^)
Ti-Al-11Si-5Cu	1A	900	1.0	9.307	0.285	0.210	616.978	42.42	32.656	4.906	318.9
	1B		1.2	9.136	0.226	0.142	597.252	38.59	40.425	2.556	166.1
Ti-Al-9Si-3Cu	2A	1000	1.0	9.121	0.191	0.250	626.128	56.69	47.754	4.758	309.2
	2B		1.2	9.112	0.164	0.247	617.540	60.10	55.561	4.274	277.8
Ti-Al-7Si-1Cu	3A	1000	1.0	9.032	0.186	0.239	672.696	56.24	48.559	4.397	285.8
	3B		1.2	8.236	0.160	0.228	636.964	58.76	51.475	3.762	244.5
Ti-Al-12Si-2Cu	4A	900	1.0	9.030	0.198	0.125	443.415	38.70	45.606	1.972	128.2
	4B		1.2	9.020	0.174	0.104	424.990	37.41	51.839	1.436	93.4
Ti-Al-13Si-6Cu	5A	900	1.0	9.226	0.240	0.182	568.096	43.13	38.442	3.607	234.4
	5B		1.2	9.054	0.216	0.171	439.132	44.19	41.917	3.085	200.5
Ti-Al-7Si-4Cu	6A	1000	1.0	9.172	0.182	0.211	667.132	53.69	50.396	3.649	237.2
	6B		1.2	9.030	0.175	0.200	617.991	53.33	51.600	3.308	215.0

**Table 3:** Quaternary composite coatings hardness property.

900 W				
Powder Composition	Sample ID	Scanning Speed (m/min)	Average Hardness (HV_0.1_)	Hardness Increase against Parent (%)
Parent	P	–	358	–
Ti-Al-11Si-5Cu	1A	1.0	396	9.60
	1B	1.2	411	12.90
Ti-Al-12Si-2Cu	4A	1.0	366	2.19
	4B	1.2	350	0.00
Ti-Al-13Si-6Cu	5A	1.0	419	14.56
	5B	1.2	528	32.20
1000 W				
Ti-Al-9Si-3Cu	2A	1.0	425	15.76
	2B	1.2	386	7.25
Ti-Al-7Si-1Cu	3A	1.0	370	3.24
	3B	1.2	389	7.97
Ti-Al-7Si-4Cu	6A	1.0	458	21.83
	6B	1.2	405	11.60

**Table 4:** Quaternary composite coatings ultimate tensile strength (UTS) property.

Specimen	Sample ID	Laser Power (W)	Scanning Speed (m/min)	Average Hardness (HV_0.1_)	UTS (MPa)
Ti-6Al-4V	Parent	–	–	358	3515
Ti-Al-11Si-5Cu	1B	900	1.2	411	4034
Ti-Al-9Si-3Cu	2A	1000	1.0	425	4168
Ti-Al-7Si-1Cu	3B	1000	1.2	389	3813
Ti-Al-12Si-2Cu	4A	900	1.0	366	3592
Ti-Al-13Si-6Cu	5A	900	1.0	528	5179
Ti-Al-7Si-4Cu	6A	1000	1.0	458	4487

**Fig. 13:**
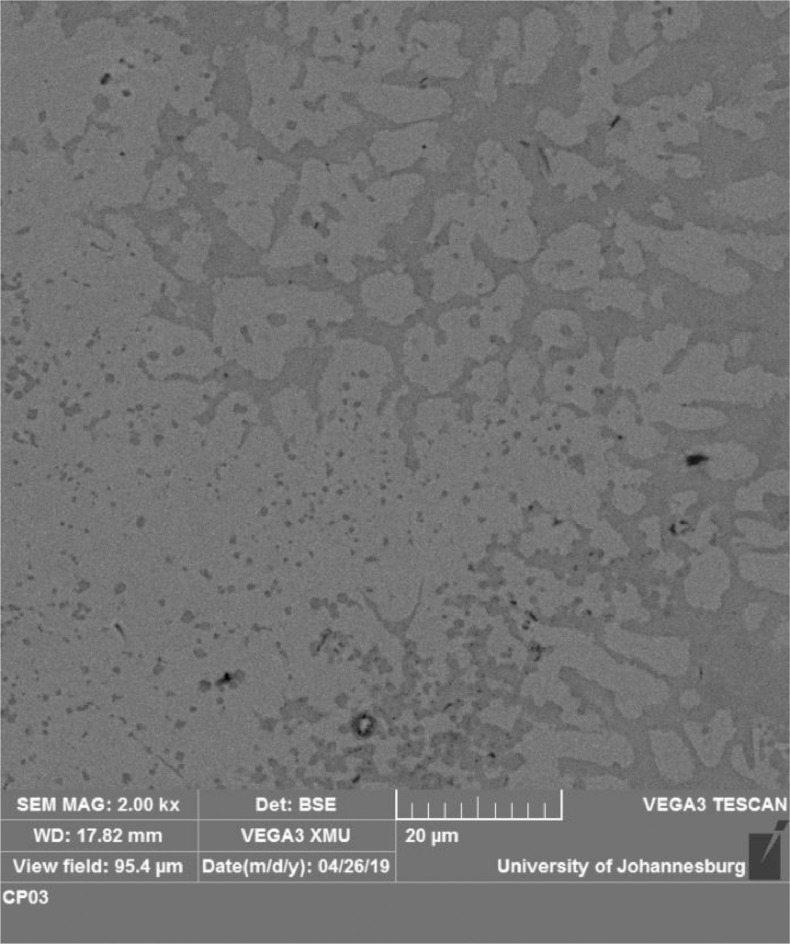
SEM image of quaternary composite coatings at 1000 W and scan velocity of 1.0 m/min.

**Fig. 14:**
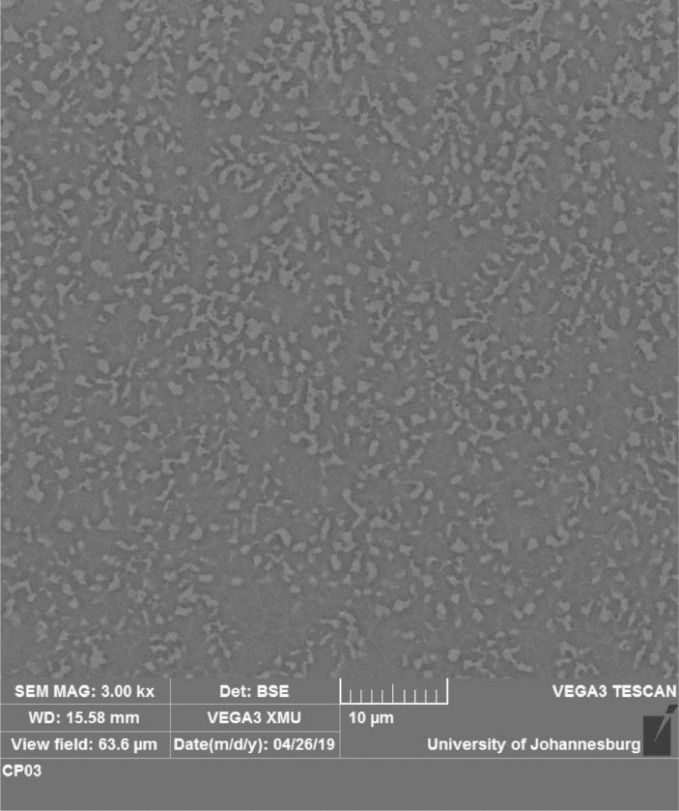
SEM image of quaternary composite coatings at 900 W and scan velocity of 1.0 m/min.

**Fig. 15:**
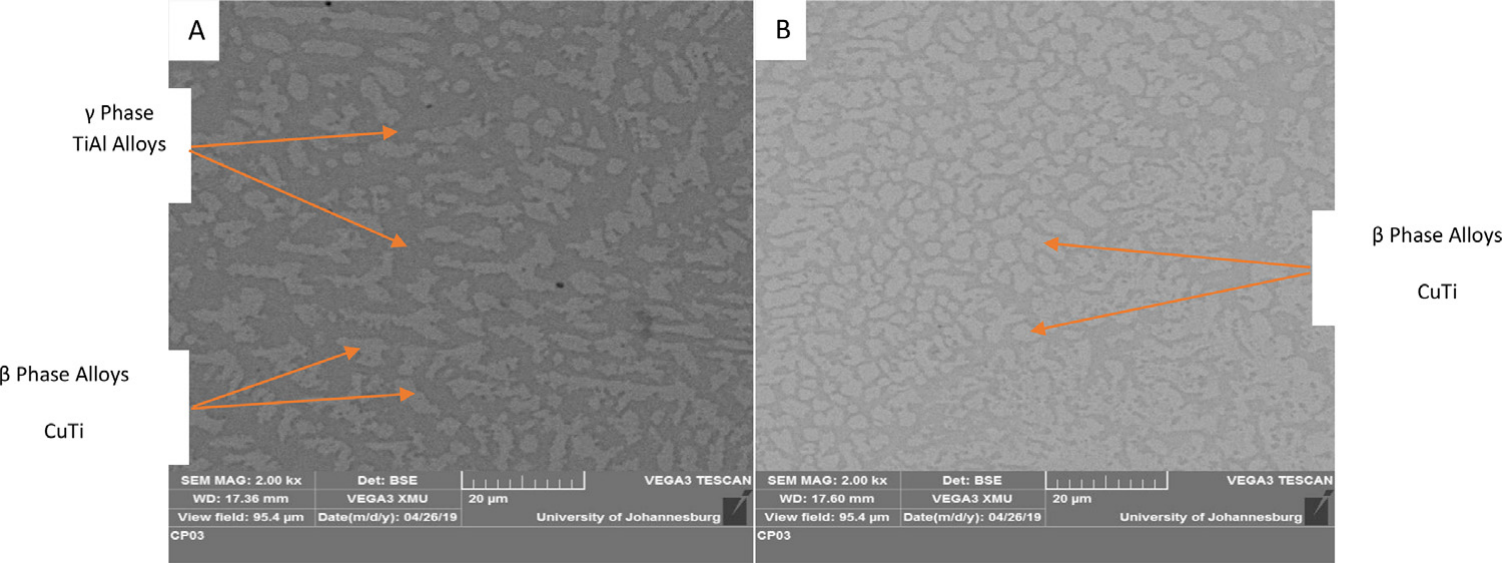
SEM image of quaternary composite coatings at (a) 900 W, 1.0 m/min and (b) 900 W, 1.2 m/min.

The significant role played by the aluminium reinforcement in the lattice structures led to the titanium-aluminides (TiAl) propagation as shown in Figs. 13–15. These are part of the phases formed in the XRD spectra shown in Figs. 10 and 11. Asides the presence of stabilizer like copper beta phase. The formation of dark grey grains (alpha grain particles) shows the presence of aluminium. Beta phase grains shows the presence of copper (brownish pigmentations) [6], [7], [8]. At increase scan velocity and laser power, dendritic grain structures were propagated showing the presence of Al-Cu binary alloy [8], [9], [10]. Moreover, intermetallic inter-dendritic eutectic phases network was formed by the combined beta and alpha phase of copper and aluminium reinforcements. There are two separate structures (inter-dendritic intermetallics and eutectic phases) which were formed in the composite coatings (Figs. 13–15) due to the presence of alpha and beta phases in the microstructure of base titanium alloy.

## 2 Experimental Designs, Materials and Method

The design of the experiment started by cutting Twelve (12) titanium alloy into dimension of 78 × 78 × 5 mm^3^ using OMAX 5500 waterjet machine. The characterization of the materials was done in order to show the microstructural evolution and all standard procedures were observed during the characterization. The etching of the samples was made possible by Nital reagent. Washing of the samples with water was followed and the samples were air dried. Desiccator in the Laboratory was used to store the etched samples. Thereafter, experimental procedure then began. The experimental procedure used average particle size of 90 μm for the reinforcements (Titanium, Aluminium and Copper powders). The mixture of the reinforcements was carried out at different weight percent via measuring scale (Shimadzu). Ti-Al-Si-Cu as follows: Ti-Al-11Si-5Cu (900 W, 1.0 m/min), Ti-Al-11Si-5Cu (1.2 m/min, 900 W), Ti-Al-9Si-3Cu (1.0 m/min, 1000 W), Ti-Al-9Si-3Cu (1.2 m/min, 1000 W), Ti-Al-7Si-1Cu (1.0 m/min, 1000 W), Ti-Al-7Si-1Cu (1.2 m/min, 1000 W), Ti-Al-12Si-2Cu (1.0 m/min, 900 W), Ti-Al-12Si-2Cu (1.2 m/min, 900 W), Ti-Al-13Si-6Cu (1.0 m/min, 900 W), Ti-Al-13Si-6Cu (1.2 m/min, 900 W), Ti-Al-7Si-4Cu (1.0 m/min, 1000 W), Ti-Al-7Si-4Cu (1.2 m/min, 1000 W). The powders were manufactured from TLS Company, Germany and the purity of the powders is 99.98%.

**Fig. 16:**
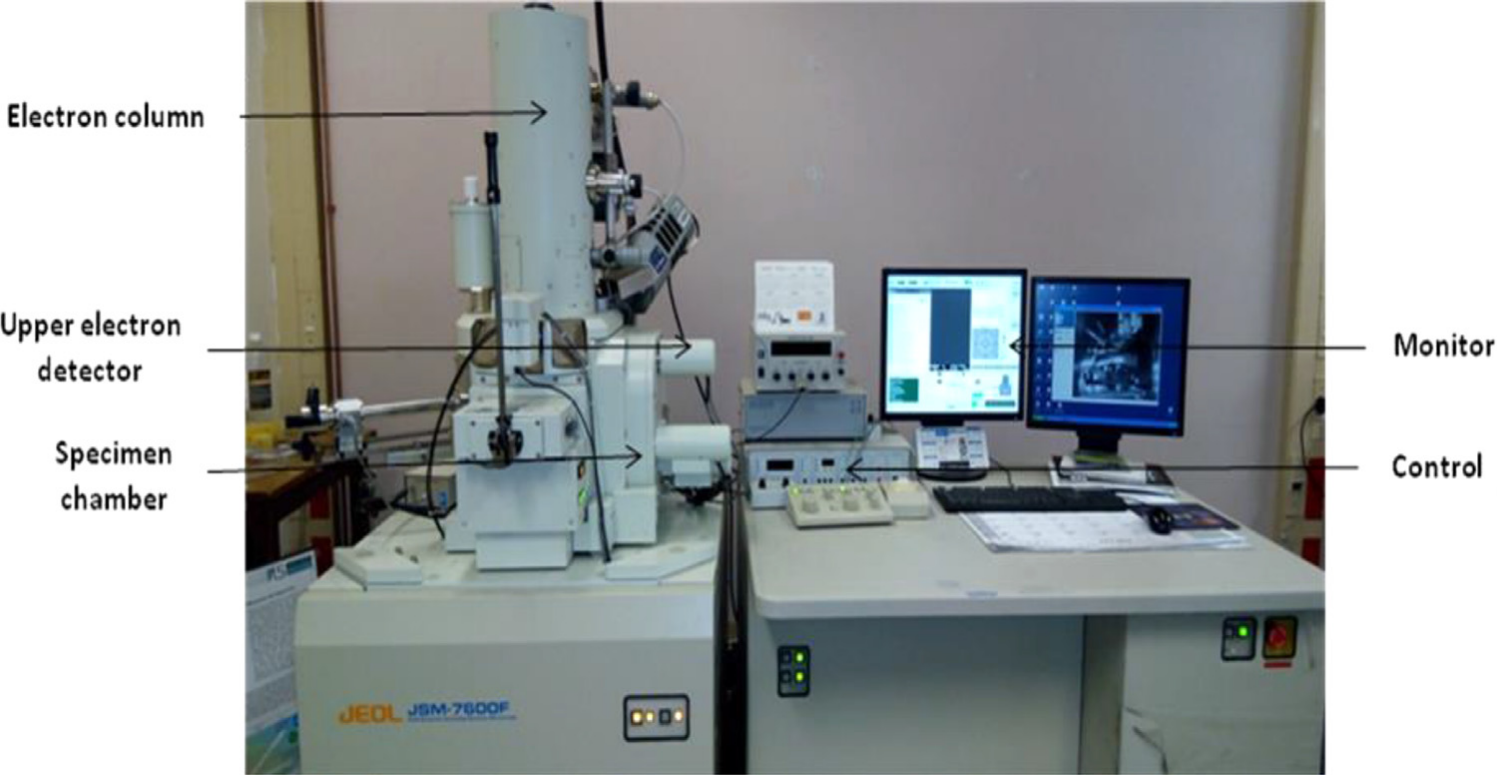
Jeol – JSM–7600F field emission scanning electron microscope.

**Fig. 17:**
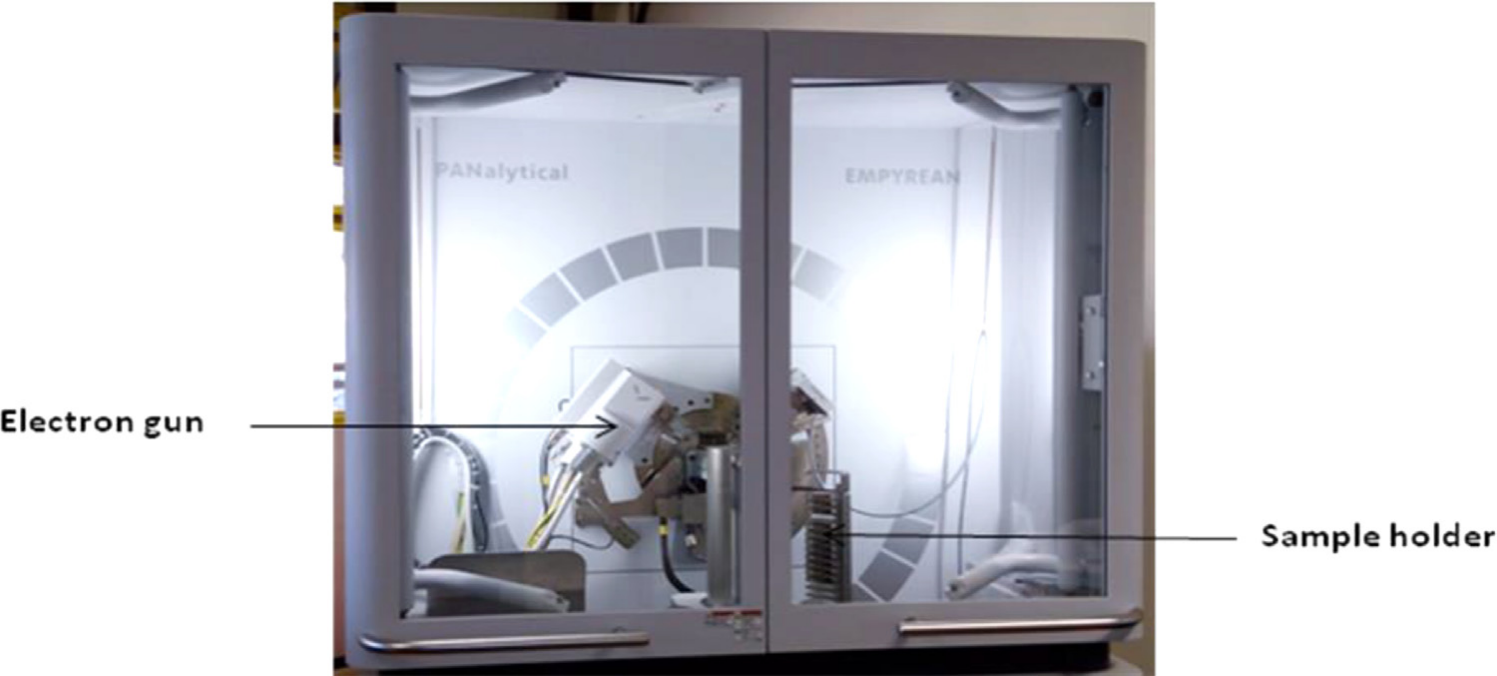
PAN analytical empyrean diffractometer.

**Fig. 18:**
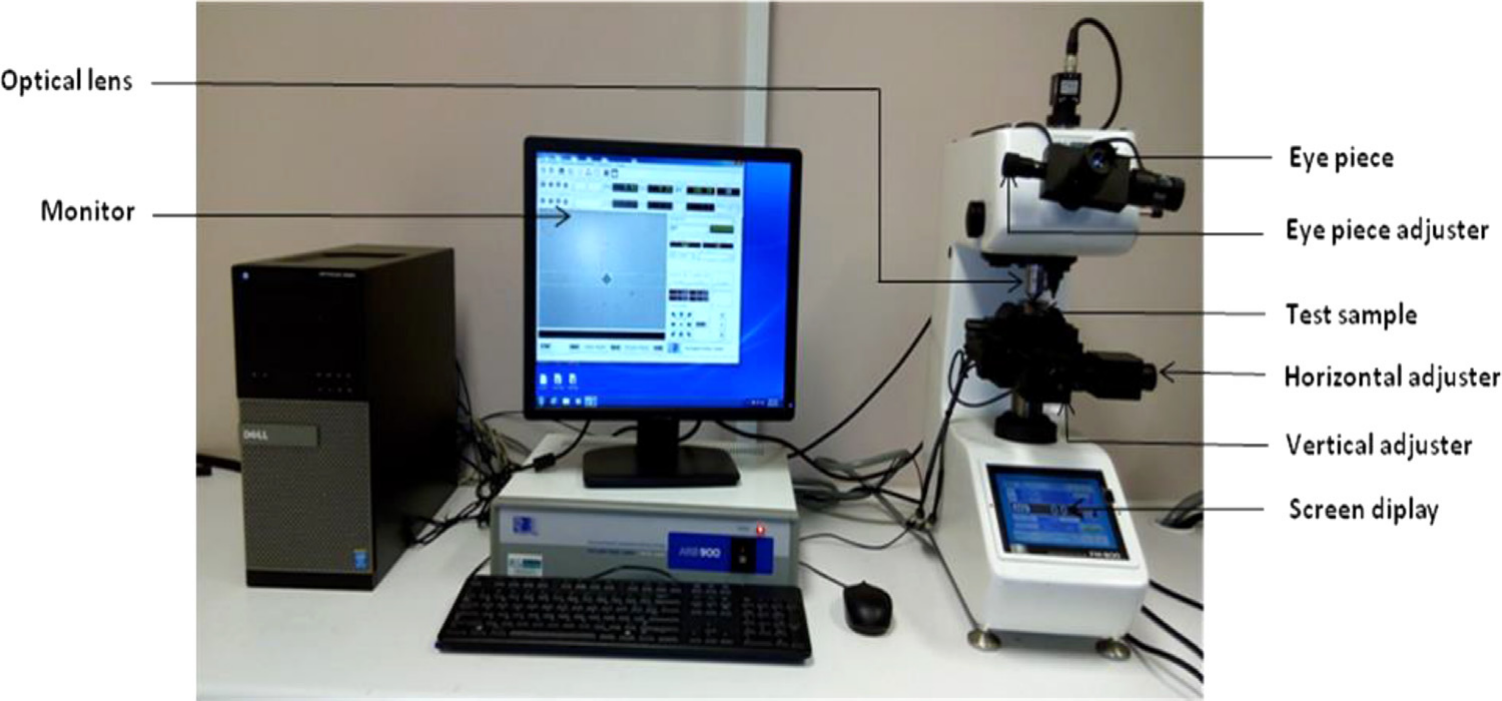
Vickers hardness tester.

The shape of the powders was manufactured as spherical. After the mixing of the reinforcements was done on the measuring scale, they were put inside a round container and paced inside a mixer (Tubular). The velocity of rotation of the mixer was set at 72 rpm and left rotating for 22 h. This was done to achieve homogenous mixture of the three reinforcements. The parameters for the experimental procedure were optimized via design of experiment. The following parameters were optimized: powder feed rate (2.5 g/min), gas shield rate (2.5 L/min), velocity of scanning (1.0–1.2 m/min), and laser power (900 – 1000 W) as shown in Fig. 1. Oxidation is not needed during the experimental procedure, so the gas (Argon) shield rate eliminates it. Argon was chosen because is cheaper than other gases. During the direct laser metal deposition (DLMD) procedure, over lapping of tracks were done at 70%. The base material distance to the tip of the nozzle was maintained at 2 mm. The procedure was performed in South Africa at National Laser Center (NLC, CSIR. 3000 W Ytterbium Laser System (YLS) was used throughout the experiment for ternary composite coatings. Though minutes pores were observed, but the optimized parameters proved microstructures homogenous of the samples. With different paper sizes, grinding and polishing were done on the coated samples. All the samples showed mirror like surfaces after polishing with diamond suspension and were rinsed in distilled water and dried in air. Characterization of all the samples considered both the surface and cross-sectional views of the microstructures. All the microstructural images of the samples were done via Olympus Microscope (BX51M) and TESCAN-X-MAX scanning electron microscopy (Fig. 16). The X-Ray Diffractometer (XRD) was done as seen in Fig. 17. The diamond indenter of the Vickers Microhardness hardness tester was used to place six indentations across the cross-section of all the samples. The indentations for hardness across the cross-section are presented in Fig. 18.

## Ethics Statement

The authors followed universally expected standards for ethical behavior in conducting and publishing scientific research.

## CRediT authorship contribution statement

**Yonas Tibebu Mekonnen:** Conceptualization, Methodology, Investigation, Formal analysis, Visualization, Validation, Writing – original draft. **Endelkachew Addis Mekonen:** Validation, Formal analysis, Visualization, Writing – review & editing. **O. Fatoba:** Conceptualization, Methodology, Investigation, Formal analysis, Visualization, Validation, Writing – original draft.

## Declaration of Competing Interest

The authors declare that they have no known competing financial interests or personal relationships which have or could be perceived to have influenced the work reported in this article.

## Data Availability

XRD spectra (Original data) (Data within article).
